# Complete mtDNA genomes of *Anopheles darlingi *and an approach to anopheline divergence time

**DOI:** 10.1186/1475-2875-9-127

**Published:** 2010-05-14

**Authors:** Marta Moreno, Osvaldo Marinotti, Jaroslaw Krzywinski, Wanderli P Tadei, Anthony A James, Nicole L Achee, Jan E Conn

**Affiliations:** 1 Wadsworth Center, Griffin Laboratory, New York State Department of Health, 5668 State Farm Road, Slingerlands, NY 12159, USA; 2 Department of Molecular Biology and Biochemistry, University of California, Irvine, CA, USA; 3 Vector Group, Liverpool School of Tropical Medicine, Liverpool, UK; 4 Instituto Nacional de Pesquisas da Amazonia, Manaus, Amazonas, Brazil; 5 Department of Microbiology and Molecular Genetics, University of California, Irvine, CA, USA; 6 Department of Preventive Medicine and Biometrics, Uniformed Services University of the Health Sciences, Bethesda, MD, USA; 7 Department of Biomedical Sciences, School of Public Health, State University of New York-Albany, Empire Plaza, Albany, NY 12201 USA

## Abstract

**Background:**

The complete sequences of the mitochondrial genomes (mtDNA) of members of the northern and southern genotypes of *Anopheles (Nyssorhynchus) darlingi *were used for comparative studies to estimate the time to the most recent common ancestor for modern anophelines, to evaluate differentiation within this taxon, and to seek evidence of incipient speciation.

**Methods:**

The mtDNAs were sequenced from mosquitoes from Belize and Brazil and comparative analyses of structure and base composition, among others, were performed. A maximum likelihood approach linked with phylogenetic information was employed to detect evidence of selection and a Bayesian approach was used to date the split between the subgenus *Nyssorhynchus *and other *Anopheles *subgenera.

**Results:**

The comparison of mtDNA sequences within the *Anopheles darlingi *taxon does not provide sufficient resolution to establish different units of speciation within the species. In addition, no evidence of positive selection in any protein-coding gene of the mtDNA was detected, and purifying selection likely is the basis for this lack of diversity. Bayesian analysis supports the conclusion that the most recent ancestor of *Nyssorhynchus *and *Anopheles*+*Cellia *was extant ~94 million years ago.

**Conclusion:**

Analyses of mtDNA genomes of *Anopheles darlingi *do not provide support for speciation in the taxon. The dates estimated for divergence among the anopheline groups tested is in agreement with the geological split of western Gondwana (95 mya), and provides additional support for explaining the absence of *Cellia *in the New World, and *Nyssorhynchus *in the Afro-Eurasian continents.

## Background

*Anopheles darlingi *is a major malaria vector in the Americas [1]. Its broad geographical distribution, ranging from southern Mexico to northern Argentina, is coupled with a high morphological, behavioral and genetic diversity [2-8]. Support for considerable genetic variation within this taxon comes from the analysis of polytene chromosomes [6], isozymes [7], as well as nuclear and mitochondrial DNA markers [8-14].

Analysis of the *An. darlingi *population structure based on the cytochrome-oxidase subunit 1 mitochondrial gene (*COI*) provided evidence in support of two significant subdivisions among *An. darlingi *populations from Central (including northwestern Colombia) and South America, with *F*_ST _values similar to those found among different species [10]. This finding was substantiated by the analysis of microsatellite markers, which revealed limited gene flow between Central America and Brazil and Peru [12]. Also, patterns of variation within the single-copy nuclear *white *gene, with five fixed non-synonymous mutations detected between the northern and southern *An. darlingi *populations (hereafter termed northern and southern genotypes) [14], were consistent with the *COI *studies. Three localities were identified in the Amazon basin where the two genotypes co-occur (Iquitos, Peru; Guayaramerín, Bolivia; Fortuna near Puerto Ayacucho, Venezuela), but no hybrid specimens were detected there.

Recent studies revealed that the distribution of the *COI *gene variants within South American populations, especially those recovered from Brazil, reflects subdivisions consistent with geographical elements, such as the Amazon River Delta and mountains in southeastern Brazil, as barriers to gene flow [13]. Significant population structure of *An. darlingi*, identified using microsatellites markers, also was reported in a malaria-endemic region of eastern Amazonian Brazil [11,12].

Overall, these findings support the hypothesis that *An. darlingi *is experiencing incipient speciation, with the northern and southern genotypes as the units of speciation [12], although other results appear to counter this hypothesis [9]. Therefore, it is critical to investigate the status of this taxon and elucidate the processes driving divergence between alternative genotypes.

The mtDNA markers are used often for phylogenetic and population genetics analyses because of ease of their amplification and high information content at different evolutionary levels, including variation within and between populations [15,16]. To date, mtDNA genomes are available for only three anopheline species, belonging to the subgenera *Anopheles *(*Anopheles quadrimaculatus *species A) [17] and *Cellia *(*Anopheles gambiae *[18] and *Anopheles funestus *[19]). Because the evolutionary lineage encompassing the subgenus *Nyssorhynchus *presumably branched prior to the divergence of the subgenera *Cellia *and *Anopheles *lineages, mtDNA of *Nyssorhynchus *species may possess different characteristics, such as altered gene order or different selection pressures, which may affect the utility of mtDNA sequences as markers for evolutionary studies. The purifying selection mechanism under which the mtDNA is assumed to evolve has been questioned recently with some reports describing positive selection acting at the mitochondrial level [20,21]. Therefore, testing the pattern of evolution of the mtDNA within the postulated incipient species, *i.e*. to examine whether the mtDNA is evolving under neutrality versus purifying or positive selection, can contribute to the understanding of *An. darlingi *differentiation.

Different approaches have been used to estimate divergence times within *An. darlingi *and to determine the most likely scenario for its diversification. Based on a single mitochondrial *COI *gene, the date of divergence was estimated to the Pleistocene, and the Pleistocene refugium was the main hypothesis to explain current distribution [10,22]. Recently, a Bayesian approach was employed to estimate divergence within other species complexes, including *Anopheles annulipes *in Australia and Papua New Guinea [23], and the Neocellia Series in the Oriental region [24]. In addition, Krzywinski *et al*. [18] calculated divergence dates of mtDNAs using a molecular clock approach with the subgenera *Anopheles *and *Cellia*, as well as with *Aedes *and between *An. gambiae *and *An. funestus*. No information exists on the time of divergence of *Nyssorhynchus*, which is hypothesized to be one of the earliest branching lineages of *Anopheles *[25]. This study tests the differentiation of the *An. darlingi *genotypes using the whole mtDNA sequence, analyses patterns of selection within this taxon, and infers a date of *Nyssorhynchus *divergence from other *Anopheles *groups.

## Methods

### Sample origin and DNA extraction

Two resting female mosquitoes were collected near local homes from Manaus, Amazonas State, Brazil (3° 08'S/60° 01' W) in August 2006 and in Central Cayo District of Belize (17° 09'N/88° 36'W) in 2008. The Belize specimens were identified morphologically following the Wilkerson and Strickman [26] illustrated morphological key and the Brazilian mosquito was identified based on both Faran and Linthicum [27] and Forattini [28] entomological keys. Mosquitoes were stored on silica gel in a sealed container or preserved in isopropanol, both at room temperature. DNA was extracted from whole mosquitoes by two different kit extractions, a DNeasy Blood and Tissue kit (Qiagen, Hilden, Germany) and Wizard Genomic DNA Purification kit (Promega, Madison, WI), following the procedures of the manufacturers.

### PCR amplification and sequencing of *Anopheles darlingi *mtDNA

The *white *gene genotype of each specimen was checked using the PCR-RFLP protocol described in Mirabello [14]. Gene amplification primers for the complete mtDNA were designed using Primer3 0.4.0 [29] based on published *An. gambiae, An. quadrimaculatus and An. funestus *mtDNA sequences (GenBank Accession NC_002084, NC_000875 and DQ146364). Each fragment overlapped upstream (5') by ~50 base pairs (bp) (see Additional file 1) and was amplified using a Ready-To-Go-PCR bead (Amersham Pharmacia/Biotech, NJ, USA) and run on a PTC-200 thermal cycler (BioRad, Inc.). Amplification conditions included an initial denaturation at 95° for 2 min, 35 cycles of denaturation at 95°C for 1 min, annealing at 58°C for 2 min and extension at 72°C for 1 min, with a final extension step at 72°C for 10 min. Amplification products were purified with the Exo-SAP-IT kit (Amersham Biosciences) and sequenced directly. Sequencing of both strands was performed at Applied Genomic Technologies Core (Wadsworth Center) on an ABI 3700 DNA automated sequencer. To ensure fidelity during amplification, each fragment was sequenced at least three times and a consensus sequence established. The regions with unclear electropherograms, from the 14,000-nucleotide position covering the whole AT-rich region, were cloned with the vector TOPO TA (Invitrogen) according to the manufacturer's recommendations.

### Sequence assembly and annotation

Sequencher 3.0 (Gene Codes Corporation) was used for sequence assembly into contigs and for proofreading sequence files. Complete consensus sequences were aligned using the ClustalW application in Bioedit v 7.0 and alignments were annotated in SEQUIN [30]. Identification of transfer RNA genes was conducted using tRNAscan-SE [31]. Protein-coding and ribosomal RNA genes were by comparison with the *An. gambiae *mtDNA sequences (GenBank accession number NC_002084).

### Genomic and evolutionary analyses

Nucleotide composition and the Relative Synonymous Codon Usage (RSU) values were calculated using the MEGA 4.1 program [32]. The GC- and AT-skews were used to determine the base compositional difference and strand asymmetry among the samples analysed [33]. Analyses of sequences also were performed with DnaSP v 4.10.9 [34] and DAMBE v 4.2.13 [35]. AT-rich region analyses and plot profiles were completed with the MacVector software (MacVector, Inc) and mreps v 2.5 [36].

To test if positive selection is acting as a force for genotype divergence, the identification of sites with an excess of amino acid replacements with selective pressure over long evolutionary time was determined. We calculated the *dN/dS *ratio (nonsynonymous and synonymous substitutions) using the entire sequence of each gene in pairwise comparisons to investigate the substitution rates in each protein-encoding gene [37]. In addition, the software PAML 3.15 was used to analyse DNA sequences with maximum likelihood methods in a phylogenetic framework [38]. CODEML is one of the programmes that can detect sites that have been under recurrent positive selection over long periods of time, *e.g*., to detect any departure from the neutrality theory in the protein sequences of *An. darlingi*. The selective pressure at the protein level was measured by ω, the ratio of nonsynonymous to synonymous rates *dN*/*dS*, with *ω *< 1, = 1, or >1 indicating conserved, neutral or adaptive evolution, respectively [39]. The measure of this ratio provides a sensitive measure of selective pressure at the amino acid level. Codon-based likelihood analysis was conducted under the random-sites model: Model M0 (one ratio) assumes constant selective pressure across codon sites and over time. Models M1a (neutral), M2a (selection), M7 (β) and M8 (β & ω) of variable selective pressure across codon sites were used to estimate selective pressure and to test for positive selection. Model M0 is the most simple and can be used to check that parameter estimates in more complex models are consistent. M0 (model = 0 NSsites = 0) was run to provide initial branch lengths, and the initial values of this tree were then used to run the other models. Because selection can act differently in some species, another approach, the branch-specific model, was also tested [40,41] to detect any evidence of positive selection in any branch of the designated tree. For this analysis the whole protein-encoding genes were used in a combined file, setting the *An. darlingi *branch as the background one (ω = 1 fixed), and allowing ω to vary in the other anopheline branches of the tree topology. In both approaches, the tree topology for each protein obtained in PAUP [42] was used as the topology to be tested under maximum likelihood.

### Phylogenetic analysis

In addition to *An. darlingi *mtDNA, the following sequences were included in the analysis: *An. quadrimaculatus *species A (GenBank L_04272), *An. funestus *(Genbank NC_008070), *An. gambiae *(NC_002084), *Aedes albopictus *(GenBank NC_006817) and *Aedes aegypti *(GenBank NC_010241). *Drosophila yakuba *(Genbank accession number NC_001322.1) was selected as the out-group [43].

Three different alignments were defined to test the phylogeny of the samples; one with the whole mtDNA sequence, the second with all the protein-encoding gene sequences concatenated and a third new data set with only 1^st ^and 2^nd ^positions of these protein-encoding genes to minimize homoplasy in the 3^rd ^codon position [44]. The software Modeltest v. 3.7 [45] was used to determine the nucleotide substitution model and the alignments followed a GTR+I+γ model (although with different rates) and were used for the subsequent phylogenetic analysis. Three different approaches were followed to reconstruct the phylogeny for posterior analyses, maximum parsimony (MP), maximum likelihood (ML) and Bayesian inference (BI). MP and ML were conducted with PAUP v 4.0b10 [42] and bootstrap values calculated with 1,000 replicates. The BI was performed employing MrBayes v 3.1.1. [46], with two simultaneous runs of 5,000,000 generations, discarding the first 25% as burn-in.

### Estimation of divergence date

Culicidae fossil records are not common, and while most of them are compressed, some specimens have been detected in amber inclusions [47]. *Anopheles *(*Nyssorhynchus*) *dominicanus *is the oldest fossil record of the Anophelinae subfamily in the New World, with controversy in the proposed age ranging from 15-20 million years ago (mya) based on foraminifera [48] and 30-45 mya based on coccoliths [49]. The deficient Diptera fossil record offers only an age estimate for the divergence between *Drosophila *and *Anopheles *at ~259.9 mya [50], and this deep external calibration might not provide reliable estimation dates, especially for the youngest nodes and for intraspecific divergences within the *An. darlingi *taxa [51]. Alternatively, Brower [52] estimated that the standard arthropod mtDNA sequence divergence is 2.3% per million years based on calculations across five arthropod genera. The limitation of applying this information to our data is that it may underestimate divergences older than 3.25 million years, because of mutation saturation [53].

A Bayesian Markov Chain Monte Carlo approach, (MCMC) available in the software BEAST v.1.4.6 [54] was performed to infer the topology and the node ages by estimating the Bayesian posterior distribution of the divergence. Following the recommendations of the authors, the uncorrelated relaxed lognormal clock was applied to estimate how clock-like our data were. The *ucld.stdev *parameter was checked, and in all partitions, the value was close to 0, indicating that the data were clock-like and that the rate of heterogeneity among species was low. Therefore, an uncorrelated lognormal relaxed clock was followed [55] and defined the rate prior to have a normal distribution with a mean of 0.1. The SRD06 model for the partition in the protein-encoding genes was selected, as it seems to provide a better fit for protein-coding nucleotide data [54]. This partition was run using the GTR with gamma and invariant sites model, the best fit with our nucleotide substitution detected by Modeltest. The Yule tree prior is the most suitable for trees describing the relationships among individuals from different species and it was selected to calculate the divergence time between taxa. Final analyses consisted of three separate MCMC runs of 20 million generations sampled every 1000 generations. Tracer v1.4 software was used to confirm the adequate mixing of the MCMC chains of the three separate runs and to check that the effective sample sizes of all the parameters were greater than 500. LogCombiner v1.4.7 was employed for combining the separate runs into one file. Finally, the maximum clade credibility tree was calculated with the mean node branch using TreeAnnotator v1.4.7. FigTree v1.2.1 was employed for visualization of the node branches.

## Results and Discussion

### Genome organization and structure

**Figure 1:**
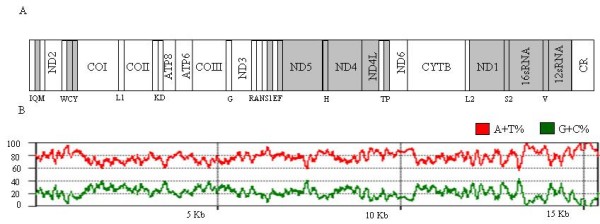
**Schematic representation of the organization of the mtDNA of *Anopheles darlingi***. A) Genes encoded by the L-strand are shaded. tRNA genes are designated by single-letter amino acid codes; L1, L2, S1 and S2 indicate tRNA- Leu (TAA), tRNA-Leu (TAG), tRNA- Ser (GCT) and tRNA- Ser (TGA). B) Graphical representation of the A+T and G+C content across the whole mtDNA. X-axis values represent the length of the genome and the Y-axis represents the percentage.

The complete mtDNAs of *An. darlingi *are 15,385 bp and 15,386 bp in length for the southern and northern genotypes, respectively. The typical 37 genes in animal mtDNA, comprising 13 protein-encoding genes, two rRNA genes (*12S *rRNA and *16S *rRNA), 22 tRNA genes and a control region are found in both genomes (Figure 1 and Table 1). DNA sequences were deposited in GenBank under accession number GQ918272 (northern genotype) and GQ918273 (southern genotype). Genes are encoded on both strands (Table 1) with some open-reading frames overlapping adjacent genes (*ATP8*/*ATP6 *and *NAD4*/*NAD4L *as reported in other insects [56]), resulting in a compact genome. The *An. darlingi *gene arrangement structure is highly conserved when compared with the mitochondrion of the other three sequenced anopheline species.

**Table 1:** Localization and features of genes in the mtDNA of southern and northern *Anopheles darlingi *genotypes.

Gene	Strand	Position	Anticodon	Size (bp)	Start codon	Stop codon
*tRNA*^*Ile*^	H	1-68	GAT	69		
*tRNA*^*Gln*^	L	66-134	TTG	69		
*tRNA*^*Met*^	H	134-202	CAT	69		
*NAD2*	H	203-1228		1026	ATT	TAA
*tRNA*^*Trp*^	H	1227-1295	TCA	69		
*tRNA*^*Cys*^	L	1295-1358	GCA	64		
*tRNA*^*Tyr*^	L	1359-1423	GTA	65		
*COI*	H	1422-2963		1542	TCG	T^a^
*tRNA*^*Leu*^	H	2959-3024	TAA	66		
*COII*	H	3027-3711		685	ATG	T^a^
*tRNA*^*Lys*^	H	3712-3782	CTT	71		
*tRNA*^*Asp*^	H	3792-3859	GTC	68		
*ATP8*	H	3860-4021		162	ATC	TAA
*ATP6*	H	4015-4695		681	ATG	TAA
*COIII*	H	4695-5481		787	ATG	T^a^
*tRNA*^*Gly*^	H	5482-5548	TCC	67		
*NAD3*	H	5549-5902		354	ATA	TAA
*tRNA*^*Arg*^	H	5902-5965		64		
*tRNA*^*Ala*^	H	5965-6030	TGC	66		
*tRNA*^*Asn*^	H	6031-6098	GTT	68		
*tRNA*^*Ser*^	L	6099-6177	GCT	79		
*tRNA*^*Glu*^	H	6166-6231	TTC	66		
*tRNA*^*Phe*^	L	6230-6296	GAA	67		
*NAD5*	L	6296-8037		1742	GTG	TA^a^
*tRNA*^*His*^	L	8038-8102	GTG	65		
*NAD4*	L	8103-9444		1342	ATG	TA^a^
*NAD4L*	L	9438-9743		306	ATG	TAA
*tRNA*^*Thr*^	H	9744-9811	TGT	68		
*tRNA*^*Pro*^	L	9812-9877	TGG	66		
*NAD6*	H	9880-10404		525	ATT	TAA
*CYTB*	H	10404-11540		1137	ATG	TAA
*tRNA*^*Ser*^	H	11539-11604	TGA	66		
*NAD1*	L	11622-12578		957	ATA	TAA
*tRNA*^*Leu*^	L	12573-12638	TAG	66		
*16S rRNA*	L	12574-13967		1394		
*tRNA*^*Val*^	L	13968-14039	TAC	72		
*12S rRNA*	L	14040-14832		793		
Control region		14833-15385, 386		555-554		

^a ^Termination codons completed via polyadenylation.

The nucleotide composition of the *An. darlingi *southern genotype is as follows: the L-strand has a GC content of 20.73% and 79.27% AT (A = 35.06%, T = 44.21%, C = 9.23% and G = 11.5%) and the H-strand has A = 44.5%, C = 16.12%, G = 7.98%, T = 31.4%. The composition of both strands of the northern genotype was not significantly different from the southern genotype. The mtDNAs of both *An. darlingi *genotypes are biased towards a high A+T content (Table 2). The overall AT content in the whole mitochondrion sequence was 78.2% and 78.1% in northern and southern, reaching 93.68% and 93.67% in the AT rich region, respectively (Figure 1). This result is similar to that obtained with other anophelines, although it is slightly lower than in the other species included in the study (Table 2). The base composition, measured by the G-skew and T-skew, *i.e*. asymmetry in nucleotide composition, showed both skews (-0.144 and -0.141, for the G skew) and (-0.028 for the T-skew), indicating the preference for these two nucleotides, a widespread characteristic of animal mtDNAs [57].

**Table 2:** Base composition of the whole mitochondrion DNA sequence of different dipterans and *Anopheles darlingi *N and S (northern and southern, respectively).

Species	Total nt	A	C	G	T	A+T	G-skew	T-skew
*Anopheles darlingi *N	15386	6189	1920	1434	5843	78.2%	-0.144	-0.028
*Anopheles darlingi *S	15385	6181	1920	1444	5840	78.1%	-0.141	-0.028
*Anopheles gambiae*	15363	6150	1989	1458	5766	77.5%	-0.154	-0.032
*Anopheles quadrimaculatus*	15455	6221	2067	1432	5735	77.3%	-0.181	-0.040
*Anopheles funestus*	15354	6178	1936	1404	5836	78.2%	-0.159	-0.028
*Aedes albopictus*	16665	6680	2014	1396	6575	79.5%	-0.181	-0.007
*Aedes aegypti*	16655	6690	2118	1380	6467	79%	-0.210	-0.016
*Drosophila yakuba*	16019	6326	1949	1481	6263	78.6%	-0.136	-0.005

### Transfer RNA and ribosomal RNA genes

The mtDNA of *An. darlingi *includes 22 tRNA genes with anticodons representing 20 different amino acids, identical to other anophelines (Table 1).

The lengths of the *12S *and *16S *rRNA genes are 793 bp and 1329 bp, respectively, identical in both genotypes, and are encoded on the L-strand. The ends of those genes were assumed to be extended to the boundaries of the flanking genes [16]. As in other anophelines, the *lrRNA *gene is flanked by the *tRNA*^*Leu *^and *tRNA*^*Val*^, while the *srRNA *gene is between *tRNA*^*Val *^and the control region. Their A+T contents were 82.54-82.39% for *lrRNA *and 79.95-79.70% for *srRNA*, which are within the range of other insects.

### Protein-coding genes

Thirteen protein-coding genes could be identified in the mtDNA of *An. darlingi*, similar to those of other Anophelinae [17-19]. Start codons in all the protein-coding genes are conserved and follow the ATN rule described previously in *Anopheles *[18], except for the *COI *gene, which has a TCG start codon characteristic of *Anopheles *[17,18,58]. Eight genes (*NAD2*, *ATP8*, *ATP6*, *NAD3*, *NAD6*, *CYTB*, *NAD4L*, *NAD1*) use the complete stop codon TAA (Table 1), whereas five genes have incomplete stop codons, TA_(*NAD4*, *NAD5*) and T_(*COI*, *COII*, *COIII*), which could be completed as a result of postranscriptional polyadenylation [59].

**Figure 2:**
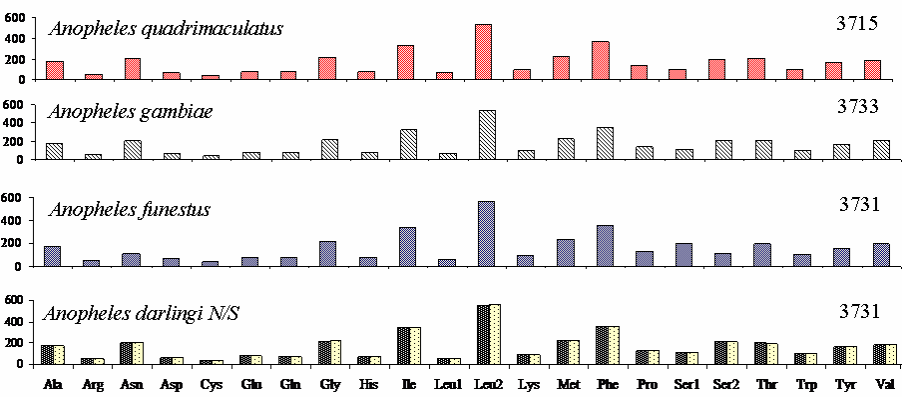
**Codon distribution in anopheline mtDNA**. The Y-axis represents the contribution of each of the codons to the total. Numbers to the left refer to the total number of codons. Amino acids names are indicated on the X-axis. N: *Anopheles darlingi *northern genotype; S: *Anopheles darlingi *southern genotype.

The codon usage of both genotypes of *An. darlingi *is nearly identical (Table 3), and the total number of non-stop codons is 3733 in both. This latter value is similar in the family Anophelinae, ranging from 3715 in *An. quadrimaculatus *to 3733 in *An. gambiae *(Figure 2).

**Table 3:** Codon usage in the southern and northern *An. darlingi *mtDNA (northern genotype in parentheses).

Codon	Count	Codon	Count	Codon	Count	Codon	Count
UUU	335 (334)	UCU	93 (94)	UAU	139 (141)	UGU	38 (37)
UUC	26 (27)	UCC	2 (3)	UAC	25 (23)	UGC	2 (3)
UUA	531 (534)	UCA	107 (108)	UAA	14	UGA	94 (95)
UUG	28 (24)	UCG	6	UAG	0	UGG	5 (4)
CUU	26 (27)	CCU	71 (72)	CAU	71	CGU	10 (9)
CUC	1 (0)	CCC	7 (6)	CAC	7	CGC	0
CUA	25 (26)	CCA	54 (55)	CAA	73 (75)	CGA	48 (47)
CUG	0	CCG	1 (0)	CAG	4 (2)	CGG	0 (2)
AUU	337 (333)	ACU	118 (115)	AAU	192 (189)	AGU	49 (53)
AUC	11 (15)	ACC	2 (4)	AAC	13 (15)	AGC	8 (6)
AUA	201 (203)	ACA	77	AAA	74 (72)	AGA	50 (49)
AUG	22 (21)	ACG	1 (3)	AAG	20 (22)	AGG	0
GUU	65 (68)	GCU	100	GAU	63 (64)	GGU	32 (34)
GUC	4 (6)	GCC	8	GAC	3 (2)	GGC	1
GUA	106	GCA	64 (65)	GAA	76 (78)	GGA	159 (161)
GUG	10 (9)	GCG	3	GAG	5 (3)	GGG	26 (20)

The sequence identity within the *An. darlingi *taxon showed a high similarity across the whole genome (Table 4), with the lowest value (98.5) for the *COII *gene. Moreover, the sequence of the protein-encoding genes is conserved in Anophelinae, while more differences are found in the Control Region (CR) [60].

**Table 4:** Percent identity based on nucleotides sequences among *An. darlingi *taxon and other anophelines.

	Identity (%)			
	
Gene	*An. darlingi *S *vs*N	*An. darlingi *S *vs An. funestus*	*An. darlingi *S *vs An. gambiae*	*An. darlingi *S *vs An. quadrimaculatus*
ND2	99.2	87.2	88.6	87.6
COI	98.6	90.6	88.6	88.2
COII	98.5	90.6	89	89.6
ATP8	98.7	91.3	90.7	91.3
ATP6	98.6	87	89.1	87.2
COIII	98.4	89.1	89.9	89.3
ND3	98.8	88.4	88.7	82.2
ND5	98.7	87.3	86.9	85.4
ND4	98.5	85.7	87.8	86.8
ND4L	99.6	90.1	88.8	87.2
ND6	99.8	85.9	87.8	88.7
CYTB	99	90.6	90	89.7
ND1	98.6	90.7	89.7	90.3
16SrRNA	99.7	92.1	93.2	94.1
12SrRNA	98.4	92.2	93.6	94.3
tRNA	100	94.9	95.8	94.3
CR	97.3	60.9	67.3	56.7

S: *An. darlingi *southern genotype; N: *An. darlingi *northern genotype; CR: control region.

### Non-coding regions

The mtDNA of *An. darlingi *contains five intergenic spacers. There are two spacers with only two nucleotides, while two others are formed by nine and 17 nucleotides. The A+T region, flanked by *12sRNA *and *tRNA*^*Ile*^, has 574 and 573 nucleotides (northern and southern, respectively), and is presumed to be the origin of DNA replication of the organelle, as reported in other insects [61]. High levels of sequence divergence were found in the CRs among anopheline species, even when low diversity was detected in other genes (Table 4). This is consistent with the CR variation observed among dipteran mtDNAs [60]. Four motifs have been described in arthropod CRs: long sequence of thymines, tandem repeat sequences, a sub-region of even higher A+T content and stem-loop structures [62]. The CR of the *An. darlingi *genotypes showed only 15 variable sites among them, including nine indels (five insertions in the northern genotype). This is surprisingly small variation, even lower than within the *An. gambiae *complex, where 52 variable positions in CR were described [63]. The anopheline CR does not have the distinct domains seen in other insects [64]. In addition, the T-stretch structure, hypothesized as the regulation of transcription of the mtDNA, is located in position 142 (17 bp of extension) and this structure is a commonly observed in the other anophelines analysed as well in other Diptera, and in different insect orders [60,64,65].

### Phylogenetic analyses

The relationships among *Anopheles *inferred using the MP, ML and Bayesian approaches are consistent with earlier reports based on mitochondrial and nuclear genes [25]. *Nyssorhynchus *was inferred as a sister taxon to *Anopheles *+ *Cellia *with high bootstrap support for each node. Inconsistent topology was obtained only in the case of rDNA sequences, which grouped *Anopheles *with *Nyssorhynchus*, likely due to non-independence of substitutions within loop regions in rDNA genes. Thus, the tree topology was consistent and employed in posterior estimation of parameters in tests of selection.

### No evidence of positive selection in the mtDNA

The Likelihood Ratio Test did not detect any sites under positive selection in any mtDNA gene. Parameter estimations of the variable ω were derived under different models for the 13 protein-encoding genes (Table 5). Analyses conducted with all the partitions were consistent with the conclusion that there is no positive selection acting on the protein-encoding genes of *An. darlingi *mtDNA, and that those are evolving by purifying selection. Even with models that allow variable selective pressure among sites (M2), ω values are distant from 1 and no indication of different selection intensities was detected. Under M8, the ω was >1 in three genes, *NAD4*, *NAD6 *and *COIII*, with less than one site with this pattern, although the comparison between models was insignificant. This could be an indication of a lack of power of the analysis, perhaps as a result of the few sequences that were available, the low divergence between them, or the lack of power of the Likelihood Ratio Test [39]. Concerns about small sample size and sequence divergence have been discussed [66]. Recently, it has been established that reasonable statistical power can be achieved with up to five genomes with divergence similar to those observed among some bacterial strains [67]. In addition, a recent study based on molecular evolution in mosquito immunity-related genes used this approach with similar sample size and with reliable results [68].

**Table 5:** Parameter estimates under five models of variable ω's among sites using PAML software.

		Parameters estimates*				
		
Gene	Codon	M0 (one-ratio)	M1a (neutral)	M2a (selection)	M7 (β)	M8 (β&ω)
*NAD1*	314	ω = 0.00843	*p*_0 _= 0.986, ω_0 _= 0.005 *p*_1 _= 0.013, ω_1 _= 1	* p*_0 _= 0.986, ω_0 _= 0.005 *p*_1 _= 0.013, ω_1 _= 1; *p*_2 _= 0, ω_2 _= 16.720	*p *= 0.040 *q *= 2.433	*p*_0 _= 0.992, *p*_1 _= 0.007 *p *= 0.047, *q *= 4.310 ω = 1
*NAD2*	341	ω = 0.00705	*p*_0 _= 0.972, ω_0 _= 0.005 *p*_1 _= 0.027, ω_1 _= 1	*p*_0 _= 0.972, ω_0 _= 0.005 *p*_1 _= 0.027, ω_1 _= 1 *p*_2 _= 0, ω_2 _= 33.683	*p *= 0.166 *q *= 17.011	*p*_0 _= 0.099, *p*_1 _= 0.003 *p *= 0.179, *q *= 19.695 ω = 1
*NAD3*	117	ω = 0.00583	*p*_0 _= 0.995, ω_0 _= 0.005 *p*_1 _= 0.004, ω_1 _= 1	*p*_0 _= 0.995, ω_0 _= 0.005 *p*_1 _= 0.002, ω_1 _= 1 *p*_2 _= 0.001, ω_2 _= 1	*p *= 0.17328 *q *= 24.05246	*p*_0 _= 0.999, *p*_1 _= 0.00001 *p *= 0.173, *q *= 24.053 ω = 1
*NAD4*	447	ω = 0.00295	*p*_0 _= 0.967, ω_0 _= 0.007 *p*_1 _= 0.032, ω_1 _= 1	*p*_0 _= 0.967, ω_0 _= 0 *p*_1 _= 0.032, ω_1 _= 1 *p*_2 _= 0, ω_2 _= 1	*p *= 0.12561 *q *= 7.98102	*p*_0 _= 0.999, *p*_1 _= 0.00001 *p *= 0.125, *q *= 7.983 ω = 1
*NAD4L*	99	ω = 0.00168	*p*_0 _= 0.980, ω_0 _= 0 *p*_1 _= 0.019, ω_1 _= 1	*p*_0 _= 0.98, ω_0 _= 0 *p*_1 _= 0.019, ω_1 _= 1 *p*_2 _= 0, ω_2 _= 88.23	*p *= 0.02510 *q *= 4.53382	*p*_0 _= 0.999, *p*_1 _= 0.00001 *p *= 0.025, *q *= 4.534 ω = 23.181
*NAD5*	576	ω = 0.01018	*p*_0 _= 0.959, ω_0 _= 0.006 *p*_1 _= 0.040, ω_1 _= 1	*p*_0 _= 0.959, ω_0 _= 0,006 *p*_1 _= 0.04, ω_1 _= 1 *p*_2 _= 0, ω_2 _= 9.342	*p *= 0.09489 *q *= 5.44623	*p*_0 _= 0.998, *p*_1 _= 0.001 *p *= 0.096, *q *= 5.702 ω = 1
*NAD6*	174	ω = 0.00034	*p*_0 _= 0.922, ω_0 _= 0 *p*_1 _= 0.077, ω_1 _= 1	*p*_0 _= 0.922, ω_0 _= 0 *p*_1 _= 0.075, ω_1 _= 1 *p*_2 _= 0.002, ω_2 _= 1	*p *= 0.11857 *q *= 99.0	*p*_0 _= 0.999, *p*_1 _= 0.00001 *p *= 0.118, *q *= 99 ω = 1.915
*ATP6*	222	ω = 0.01750	*p*_0 _= 0.966, ω_0 _= 0.012, *p*_1 _= 0.033, ω_1 _= 1	*p*_0 _= 0.966, ω_0 _= 0.01 *p*_1 _= 0.013, ω_1 _= 1 *p*_2 _= 0.02 , ω_2 _= 1	*p *= 0.12790 *q *= 5.29443	*p*_0 _= 0.999, *p*_1 _= 0.00001 *p *= 0.127, *q *= 5.295 ω = 1
*ATP8*	53	ω = 0.03705	*p*_0 _= 0.999, ω_0 _= 0.037 *p*_1 _= 0.000, ω_1 _= 1	*p*_0 _= 1, ω_0 _= 0.037 *p*_1 _= 0, ω_1 _= 1 *p*_2 _= 0, ω_2 _= 1	*p *= 0.63306 *q *= 15.13648	*p*_0 _= 0.999, *p*_1 _= 0.00001 *p *= 0.633, *q *= 15.138 ω = 1
*COI*	512	ω = 0.00343	*p*_0 _= 0.997, ω_0 _= 0.003 *p*_1 _= 0.002, ω_1 _= 1	*p*_0 _= 0.997, ω_0 _= 0.003 *p*_1 _= 0, ω_1 _= 1 *p*_2 _= 0.002, ω_2 _= 1	*p *= 0.00827 *q *= 0.29316	*p*_0 _= 0.999, *p*_1 _= 0.00001 *p *= 0.011, *q *= 0.524 ω = 1
*COII*	228	ω = 0.00761	*p*_0 _= 0.999, ω_0 _= 0.007 *p*_1 _= 0, ω_1 _= 1	*p*_0 _= 1, ω_0 _= 0.007 *p*_1 _= 0, ω_1 _= 1 *p*_2 _= 0, ω_2 _= 31.418	*p *= 0.05952 *q *= 5.03366	*p*_0 _= 0.999, *p*_1 _= 0.00001 *p *= 0.059, *q *= 5.033 ω = 1
*COIII*	262	ω = 0.00865	*p*_0 _= 0.984, ω_0 _= 0.005 *p*_1 _= 0.015, ω_1 _= 1	*p*_0 _= 0.984, ω_0 _= 0.005 *p*_1 _= 0.015, ω_1 _= 1 *p*_2 _= 0, ω_2 _= 68.73	*p *= 0.01261 *q *= 0.42587	*p*_0 _= 0.999, *p*_1 _= 0.00001 *p *= 0.0125, *q *= 0.422 ω = 2.891
*CYTB*	378	ω = 0.00516	*p*_0 _= 0.988, ω_0 _= 0.002 *p*_1 _= 0.011, ω_1 _= 1	*p*_0 _= 0.988, ω_0 _= 0.002 *p*_1 _= 0, ω_1 _= 1 *p*_2 _= 0.01, ω_2 _= 1	*p *= 0.01168 *q *= 0.44511	*p*_0 _= 0.994, *p*_1 _= 0.0051 *p *= 0.012, *q *= 0.583 ω = 1

*Pi is the proportion of sites to an ω category or to a β distribution with parameters *p *and *q*, depending on the model.

### Estimates of time of most recent common ancestor

The analyses performed with all mtDNA genes pushed back the split between Culicinae (subgenus *Stegomyia*) and Anophelinae (genus *Anopheles*) to 190 mya, in the Early Jurassic. The *Anopheles *radiation took place during the Early Cretaceous (130 mya), and the most recent ancestor of the *Anopheles *and *Cellia *subgenera is estimated at 107 mya. Finally, the split within the *Cellia *subgenus (*An. gambiae *and *An. funestus*) was estimated at 82 mya. Alternatively, the analysis of the protein-coding genes provides earlier divergence dates, and although they correspond to the same geological periods, the nodes are dated more recently in agreement with an older origin of the mosquitoes in the Jurassic [69,70]. A recent study based on six nuclear genes and morphological characters provides further support for a Jurassic origin of mosquitoes although the estimated divergence between *Anopheles *and *Cellia *was more recent (~43 mya) than the dates derived from our study [71]. The different evolutionary histories of the molecular markers (mtDNA vs nDNA), besides incomplete species sampling, may explain the apparent incongruence between the dates.

**Figure 3:**
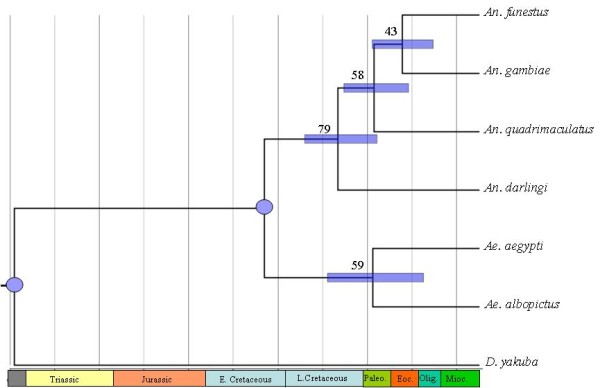
**Chronogram for the Subfamily Anophelinae and Culicinae**. Node positions indicate mean estimated divergence times, and node bars indicate the 95% confidence intervals. The circles represent the calibration points: *Drosophila*-*Anopheles *259.9 MY [50], and *Anophelinae*-*Culicinae *120 MY [71].

The inclusion of an additional calibration point of the divergence of Anophelinae and Culicinae estimated to 120 mya in the Early Cretaceous [72] was incorporated in the analysis, providing a node age divergence of the *Nyssorhynchus*-*Anopheles *of 79 mya. The process of separation between South America and Africa began in the Permian and the last connection by land was estimated to exist ~95 mya. In addition, the split between *Anopheles*-*Cellia *took place 58 mya (Figure 3). Altogether, these data provide estimates for the radiation of the subgenus *Cellia *concordant with the loss of land bridges between Africa-Europe and Europe-North America (through Greenland), that may explain the current distribution of the *Cellia *subgenus, absent in the Americas. *Anopheles *distribution across the American continent, in agreement with the radiation dates derived from this study, is sustained from a latitudinal migration and subsequent radiation from the north to the south. The large confidence intervals of both analyses include congruent dates that fit with the above explanation, although these estimates should be read carefully, since the use of few calibration points could provide weak estimates of divergence [73].

The study of incipient species has been of paramount importance in the understanding of speciation in different organisms [74,75]. Even though there is strong evidence for diverging genotypes in *An. darlingi*, its mtDNA does not sustain this level of variance. The persistence of permeable barriers that permit exchange of genetic information within genotypes could preclude the detection of the differentiation. Questions focused on reproductive isolation among the *An. darlingi *genotypes need to be addressed, as well as assortative mating features between co-existing populations. Taxa with a recent divergence time are expected to share ancestral variation in high proportions, which may confound the reconstruction of their historical relationships. Furthermore, it is likely that young species, with a recent divergence process, have fixed differences only at genes directly responsible for speciation. The major African malaria vector, *An. gambiae*, has incomplete barriers to gene flow, where divergent selection is acting on local regions of the genome, and gradually increasing the divergence of the populations until gene flow disappears between them [76,77]. Further support for this form of divergence was provided with the "speciation island model", which suggests small regions with high differentiation in the genome of this taxon [77,78].

The current studies focused on deciphering the complete genome of *An. darlingi *will provide new elements and tools to further evaluate the speciation of this taxon. Subsequently, the potential identification of genes affecting speciation should improve the surveillance and management of malaria programs in the Americas and help to elucidate the different patterns of malaria transmission involving *An. darlingi*.

In addition, the radiation of anophelinae species underscores the significance of these studies together with the origin of malaria parasites. The link between the anophelines in the New World and malaria parasites is still controversial [79-81]. If human *Plasmodium *parasites were transferred to the Americas by European colonialists in post-Colombian times, then the contact between neotropical malaria vectors and protozoan is very recent. This supports the hypothesis that the susceptibility of mosquitoes to malaria infection is a basal characteristic and further investigations should be conducted to elucidate the refractoriness to this parasite in non-malaria vector species.

## Conclusions

The lack of conclusive evidence of speciation at the mtDNA level demands further investigation of other markers, such as nuclear genes that can reflect differences in ecological preferences within *An. darlingi*. The divergence dates estimated for the subgenus *Nyssorhynchus *provide valuable information, which, although there is a need to be cautious, because the constraints of the method gave an approximation of the radiation of this group. Finally, it seems that purifying selection is predominant at the mitochondrial level, and the differences observed within the taxon may not be a consequence of that force.

## Abbreviations

The abbreviations used in this work were: *COI*, *COII*, *COIII*: cytochrome oxidase subunit I, II, and III protein genes; *CYTB*: cytochrome b gene; *ATP6*, *ATP8*: ATP synthase subunit 6 and 8 genes; *NAD1*, *NAD2*, *NAD3*, *NAD4*, *NAD4L*, *NAD5*, *NAD6*: NADH dehydrogenase subunit 1-6, 4L genes.

## Competing interests

The authors declare that they have no competing interests.

## Authors' contributions

MM and OM participated in the design of the study, performed molecular and genetic analysis and wrote the manuscript. JK and AAJ advised on the analysis and assisted in preparing the manuscript. WPT and NLA provided samples used in this study and helped to draft the manuscript. JEC conceived and supervised the study, and assisted in the writing of the manuscript. All authors read and approved the final manuscript.

## Supplementary Material

Additional file 1 Sequence and position of the primers used for the amplification of the mtDNA of *Anopheles darlingi*. Click here for file
