# Supplementary material for: Complete mtDNA genomes of Anopheles darlingi and an approach to anopheline divergence time
Source: Malar J. 2010 May 14;9:127. doi: 10.1186/1475-2875-9-127 (PMC2877063; doi:10.1186/1475-2875-9-127)
Supplement: Additional file 1 — Sequence and position of the primers used for the amplification of the mtDNA of Anopheles darlingi. [file 1475-2875-9-127-S1.DOC]

**Additional files**

**Additional file 1 - Sequence and position of the primers used for the amplification of the mtDNA of *Anopheles darlingi***.

|  | |
| --- | --- |
| **Nucleotide position** | **Primer sequence *5’-3’*** |
| 8-561 | F: TGCCTGATGAAAAGGATTACCTTG  R: CCTTCCATTACTTCAGGGAACCA |
| 539-1498 | F: TGGTTCCCTGAAGTAATGGAAGG  R: ATTCCAGCTCAAGCTCCAAAAAT |
| 945-1796 | F: GAGGATTGCCTCCATTTTTAGGA  R: AGTTCATCCTGTTCCAGCTCCAT |
| 1768-2750 | F: TAGAAAATGGAGCTGGAACAGGA  R: TCTGGAAAATCTGAATAACGTCGAG |
| 2420-3275 | F: CCCAGCTATACTATGAGCCTTTGG  R: AATCGTAATGAAGGGAACGCAAT |
| 3028-3881 | F: ATGGCAACATGAGCAAATTTAGG  R: AATTGGAGCTATTTGTGGGATTAAAAA |
| 3549-4499 | F: AACACCAGGCCGACTAAATCAAC  R: ATCGCACTGCTAAAGTTCCTGGT |
| 4434-5411 | F: TGCCATTTATGGTATGCATTGAA  R: GCAGCTTCAAATCCAAAATGATG |
| 5035-5899 | F: GGAATAACATGACCTCCAGTAGGAGT  R: GGAATAACATGACCTCCAGTAGGAGT |
| 5863-7258 | F: ACCATGAATGAAATCAAGGAGCA  R: GCTGGATTAGGAGCAAATTTTGAGT |
| 7236-8527 | F: TCAAAATTTGCTCCTAATCCAGCTA  R: GCTCATGGGTTATGTTCATCTGG |
| 8505-9540 | F: CCAGATGAACATAACCCATGAGC  R: TTTTGTGTTTGTGAAGGAGTGTTAGG |
| 9513-10664 | F: GACCTAACACTCCTTCACAAACACAA  R: CCATTTGCGTGACAAATTCGTAG |
| 10461-11445 | F: AGTAGATTTACCCGCACCATCAAA  R: TGGATCTTCTACTGGTCGTGCTC |
| 11200-12156 | F: CCAGTTCATATTCAACCCGAATG  R: GTTACGAGCGGTTGCTCAAACTA |
| 11945-12845 | F: CTTCGGCAAAATCAAAAGGAGTT  R: CGACCTCGATGTTGGATTAAGAG |
| 12180-13171 | F: ATTTGAAGATCACCCCGCAATTA  R: GGCTGGTATGAATGGTTGAATGA |
| 13154-14647 | F: CAACCATTCATACCAGCCTTCAA  R: CTGCGGTTATACAAATGATGCAA |
| 14110-14977 | F: GGCGATGTGTGCATGTTTTAGAG  R: AAAATAGGGGGTTTATGGGGAGA |
